# Polymorphic Transformation and Dislocation Regulation in MoS_2_ Enabled by Electric Field Postprocessing for Enhanced Electromagnetic Wave Absorption

**DOI:** 10.1007/s40820-026-02334-1

**Published:** 2026-08-12

**Authors:** Xiangdong Wang, Zijing Li, Shengchong Hui, Hongjing Wu, Jiaming Wen, Limin Zhang

**Affiliations:** 1 State Key Laboratory of Porous Metal MaterialsSchool of Physical Science and Technology, Northwestern Polytechnical University, Xi’an, 710072 People’s Republic of China; 2 Shenzhen Research Institute of Northwestern Polytechnical University, Sanhang Science &Technology Building, No. 45th, Gaoxin South 9th Road, Nanshan District, Shenzhen, 518057 People’s Republic of China

**Keywords:** Electrical field postprocessing, MoS_2_, Polarization loss, Electromagnetic wave absorption

## Abstract

**Highlights:**

- A direct current electric field postprocessing strategy enables precise control over the electronic structure and phase ratio of MoS_2_.
- Dislocation induced dipole arrays synergize with sulfur vacancies to enhance dipole polarization.
- Synergizing multiple loss mechanisms at 6 V for 10 min delivers an effective absorption bandwidth of 6.72 GHz, a 440% enhancement over the untreated sample.

**Abstract:**

Electric field modulation offers a non-contact route to tune electromagnetic wave absorption (EWA) by controlling carrier behavior. However, current in-situ electric field modulation strategies are often hindered by multi-physics factors during synthesis, limiting a deeper understanding of the decoupled mechanism of the electric field. Here, we report a postprocessing strategy that employs direct current electric field to induce *d*-orbital electron migration, triggering the 2H to 1T-phase transition in MoS_2_, accompanied by dislocation generation. On one hand, the increased 1T-phase content optimizes the conductive loss. Meanwhile, the Fermi level mismatch at 2H/1T interfaces creates electron accumulation regions that drive polarization loss. On the other hand, positive and negative charges accumulate on opposite sides of the dislocation lines, forming ordered equivalent dipole arrays, markedly boosting polarization. After treatment at 6 V for 10 min, MoS_2_ achieves an effective absorption bandwidth of 6.72 GHz at 2.20 mm, a 440% enhancement relative to the untreated sample. This improvement exceeds the typical 150% to 350% enhancement achieved by most field modulation strategies. This work demonstrates that the electric field postprocessing strategy effectively tailors the phase ratio, defects, and EWA performance of MoS_2_, offering a pathway for the design of advanced electromagnetic wave absorbers.

**Figure:**
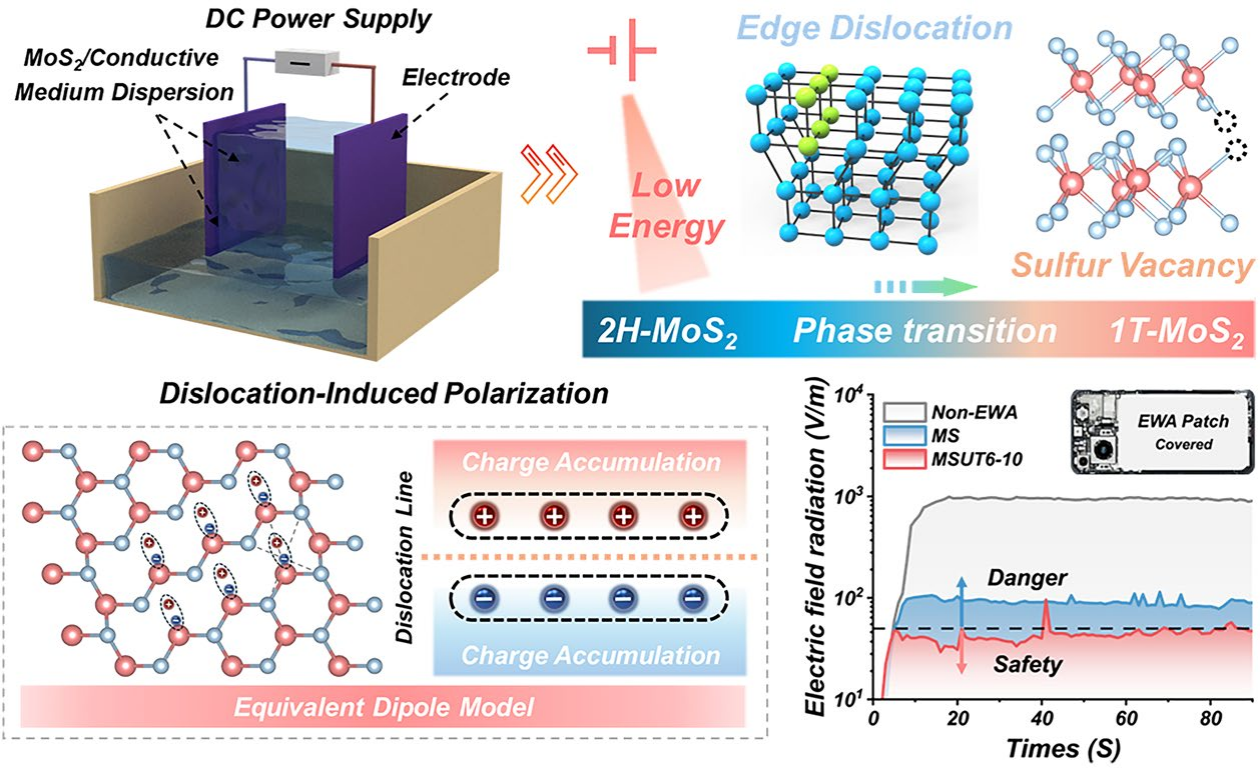

**Supplementary Information:**

The online version contains supplementary material available at 10.1007/s40820-026-02334-1.

## Introduction

Two-dimensional transition metal dichalcogenide MoS_2_ has emerged as a research focus in numerous cutting-edge fields owing to its tunable electronic structure and rich interfacial effects [1–5]. For electromagnetic wave absorption (EWA), its performance is essentially determined by the microscopic electronic configuration. The semiconducting 2H phase features a trigonal prismatic coordination. Unique d_z_^2^ orbital hybridization and symmetry breaking yield a moderate intrinsic permittivity, which facilitates impedance matching but is limited in terms of dielectric loss by its low carrier concentration [6–9]. In contrast, the metallic 1T phase possesses octahedral coordination. *d*-orbital splitting produces partially filled t_2g_ band, delivering superior electrical conductivity. Although high conductivity enhances attenuation through conductive loss, it simultaneously triggers a pronounced skin effect that intensifies electromagnetic reflection and leads to severe impedance mismatch [10–15]. Accordingly, actively regulating the phase fraction and intrinsic defects of MoS₂ to tune electronic structure transitions for the precise optimization of microwave absorption performance remains a key scientific challenge.

To address these limitations, current efforts have predominantly focused on static modification strategies, namely ion doping [16] and heterojunction engineering [17]. Although these approaches can introduce additional polarization centers or conductive pathways to moderately boost EWA [18, 19], they possess unavoidable intrinsic limitations. Ion doping often suffers from limited universality and ion mismatch, which easily triggers phase separation. Meanwhile, heterojunction construction typically requires complex, multi-step syntheses, precluding simultaneous and precise control over intrinsic electromagnetic parameters. External field postprocessing (e.g., ion intercalation [20], low-temperature plasma processing [21]) offers a promising alternative by inducing lattice distortion via energy injection [22], circumventing complex chemical procedures while enabling optimized absorption performance. However, these methods also face challenges: ion intercalation may introduce impurity species that disrupt charge transport, and low-temperature plasma treatment is generally confined to surface modification [23]. Consequently, the development of a universal and highly tunable external field postprocessing strategy that enables precise modulation from electronic structure to phase composition is urgently needed.

Against this backdrop, electric field postprocessing has emerged as a particularly promising external field modulation strategy [24], distinguished by its mild operating conditions and precise controllability. Upon direct current (DC) electric field, this approach induces *d*-orbital electron migration in Mo atoms within MoS_2_, driving a coordination transformation from the semiconducting 2H phase (trigonal prismatic) to the metallic 1T phase (octahedral), while simultaneously constructing a three-dimensional conductive network. In parallel, the introduction of defects such as vacancies and dislocations [25] during the phase transition, along with polarization effects at the 2H/1T interface, contributes to enhanced electromagnetic wave attenuation. This electronic-structure-level modulation establishes a physical foundation for optimizing phase composition and advancing absorption performance. Guided by this rationale, we selected hydrothermally synthesized nanoflower-like MoS_2_ as the model system. Its three-dimensional architecture not only amplifies interfacial polarization and conductive loss but also integrates synergistically with electric field postprocessing, offering an ideal platform to systematically explore how applied voltage and treatment duration govern phase evolution and electromagnetic response.

We propose a postprocessing modulation strategy based on a DC electric field, which enables external field control over the electronic structure of MoS_2_, thereby tailoring charge distribution and carrier concentration to synergistically enhance both polarization and conduction loss. In this study, MoS_2_ powder was uniformly mixed with polyvinylpyrrolidone (PVP) and sodium chloride electrolyte solution, followed by electric field postprocessing with systematically adjusted applied voltages and treatment durations. Under optimal conditions (6 V, 10 min), the 1T-phase fraction nearly doubled, and an effective absorption bandwidth (EAB) of 6.72 GHz was achieved at a matching thickness of 2.20 mm. Such performance improvement stems from electric field induced lattice distortion driven by *d*-orbital electron migration of Mo atoms, which accelerates the phase transition from 2H to 1T. Meanwhile, abundant 2H/1T interfaces are formed, accompanied by the generation of sulfur vacancies and dislocations. The interfaces act as space-charge accumulation centers, inducing interfacial polarization. Dislocations induce separation of positive and negative charge centers, forming equivalent dipole arrays along the dislocation lines [26]. This effect facilitates charge accumulation on opposite sides of the dislocation lines, making the equivalent dipoles more susceptible to polarization loss under an external electric field, which together with sulfur vacancies further strengthens dipole polarization. Simultaneously, the three-dimensional conductive network established by the 1T phase greatly elevates conductive loss. This work demonstrates that electric field postprocessing actively tailors phase fraction and defects via lattice distortion, enabling precise control over EWA. These findings establish a theoretical and experimental foundation for the design of field-tunable intelligent wave-absorbing materials.

## Experimental Section

### Materials

Ammonium molybdate tetrahydrate ((NH_4_)_6_Mo_7_O_24_·4H_2_O, AR), thiourea (CH_4_N_2_S), polyvinylpyrrolidone (PVP), sodium chloride (NaCl), *N*, *N*-dimethylformamide (DMF), and deionized water were all provided by Shanghai Maikelin Biochemistry Co., Ltd. All chemical reagents were used as received without further purification.

### Preparation of MoS_2_

MoS_2_ was synthesized via a hydrothermal method. Specifically, 2 mmol of ammonium molybdate tetrahydrate and 30 mmol of thiourea were dissolved in 70 mL of deionized water under magnetic stirring for 30 min to ensure homogeneous mixing. The resulting solution was transferred into a 100-mL stainless steel autoclave lined with polytetrafluoroethylene (PTFE), and heated at 200 °C for 18 h. After the reaction, the product was alternately washed three times with deionized water and absolute ethanol, then dried in a vacuum oven at 60 °C for 18 h. The obtained MoS_2_ sample was designated as MS.

### Electric Field Postprocessing of MoS_2_

Electric field postprocessing of MoS_2_ was performed using a DC power supply with an adjustable voltage range of 0–36 V. In a typical procedure, 0.1 g of PVP and 0.02 mol of sodium chloride were dissolved in 20 mL of deionized water under magnetic stirring for 30 min. Subsequently, 0.05 g of MoS_2_ and 20 mL of DMF were added sequentially, and the mixture was stirred for another 10 min to obtain a homogeneous suspension. The suspension was then subjected to DC electric field treatment with voltage ranging from 4 to 7 V and treatment durations ranging from 0 to 30 min. After the treatment, the suspension was separated by centrifugation, alternately washed three times with deionized water and absolute ethanol, and finally dried in a vacuum oven at 60 °C for 24 h. The resulting samples were denoted as MSUTX- Y, where X and Y represent the applied voltage and treatment duration, respectively.

### Characterization of Electromagnetic Parameters

Electromagnetic parameters of all samples were measured using an MS46322B vector network analyzer (VNA, Anritsu, Japan). Each as‑treated MoS_2_ sample was mixed with paraffin wax at a mass ratio of 1:1 and compressed into a coaxial ring (outer diameter *Φ*_out_ = 7.00 mm, inner diameter *Φ*_in_ = 3.04 mm). The complex permittivity and permeability were recorded over 2–18 GHz via the coaxial method. For samples prepared under different voltages and treatment durations, all measurements were carried out under identical conditions with a consistent filler loading of 50 wt%.

## Results and Discussion

### Controlled Synthesis and Construction of 1T-Phase MoS_2_ via Electric Field Postprocessing

**Fig. 1:**
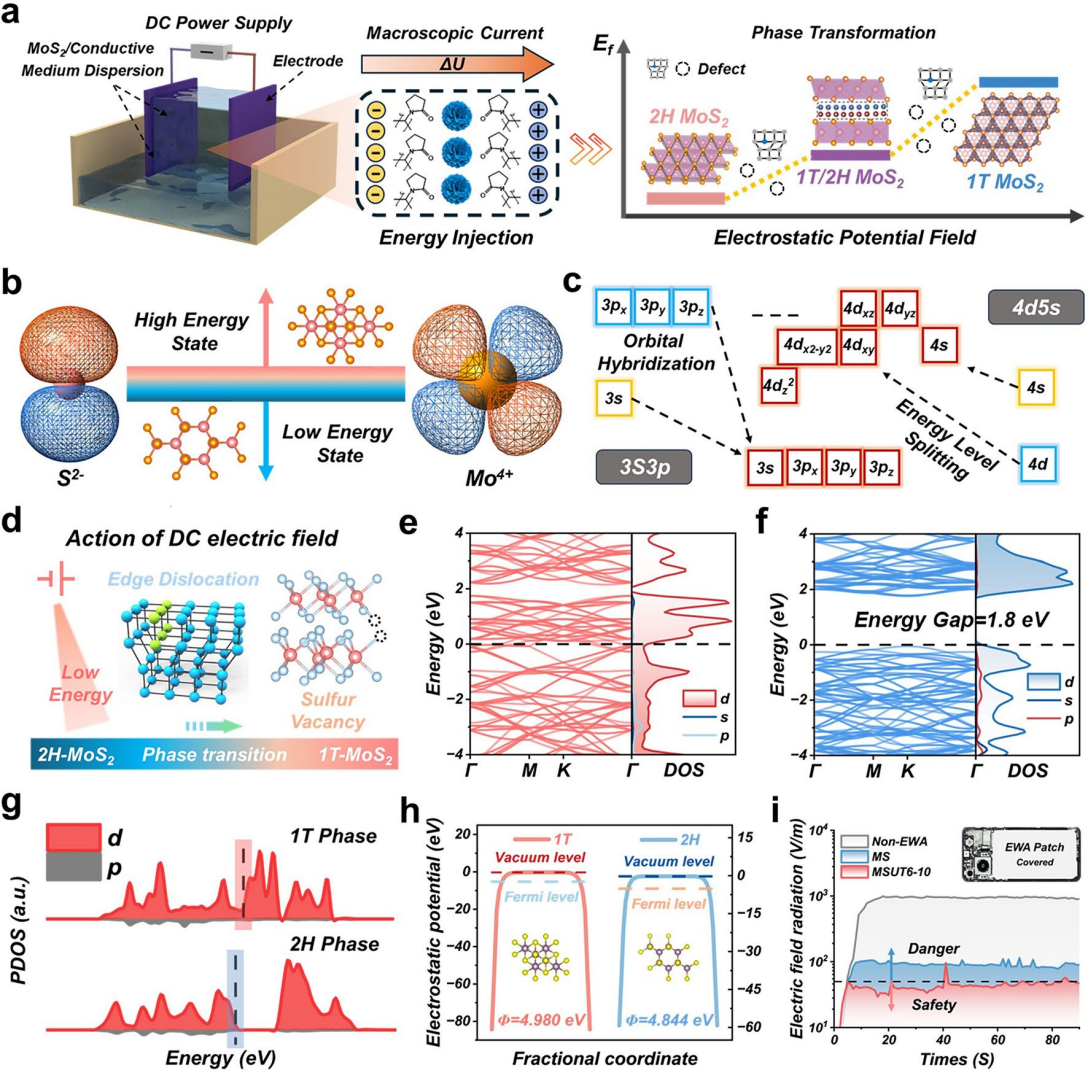
**a** Schematic illustration of the electric field postprocessing process; **b** schematic diagram of molecular energy levels and charge transfer; **c** Schematic diagram of orbital hybridization and energy level splitting; **d** schematic illustration of the application of a DC electric field; **e**, **f** simulated band structures of 1T-MoS_2_ and 2H-MoS_2_; **g** schematic DOS for 1T-MoS_2_ and 2H-MoS_2_; **h** work functions of 1T-MoS_2_ and 2H-MoS_2_; **i** radiation values detected by electronic communication devices under different conditions

Three-dimensional nanoflower-like MoS_2_ precursors were synthesized via a hydrothermal method and subsequently dispersed into a stable suspension using (PVP) as a surfactant in a sodium chloride electrolyte. This suspension was subjected to postprocessing under a controlled DC electric field, wherein precise modulation of voltage and processing time enabled directed control over charge distribution and transport behavior, illustrating a continuous process from macroscopic energy injection to microstructural reconstruction (Figs. 1a and S1, S2). This strategy induces lattice distortion within the material, generating defects such as vacancies and dislocations through mechanisms involving local electronic density of states modulation and band restructuring. The microscopic driving force of electric field induced electron transfer is elucidated in Fig. 1b from the perspective of S and Mo ion coordination, depicting directional charge transfer between ions and the accompanying energy variation. Figure 1c schematically represents the hybridization of S3*s* and 3*p* orbitals and their interaction with Mo valence orbitals (4*d*,5*s*). As the coordination environment transitions from the trigonal prismatic configuration of the 2H phase to the octahedral configuration of the 1T phase, electrons in Mo 4*d* orbitals migrate to higher-energy states such as 4*d*_xz_ and 4*d*_yz_, intensifying the Jahn–Teller effect and thereby inducing lattice distortion to achieve a stable electronic configuration (Figs. S3–S5) [27].

Based on this understanding, gradient experiments were designed to systematically investigate the effects of voltage and processing time on dielectric response, with particular focus on their synergistic interplay. Under the optimal condition of 6 V for 10 min, the best-performing MoS_2_ sample was obtained and is hereafter denoted as MSUT6-10 (MoS_2_-6 V-10 min). The schematic illustration of the DC electric field application (Fig. 1d) further elucidates the defect engineering mechanisms accompanying the phase transition. Owing to its low-energy and sustained nature, the electric field promotes the formation of dislocations and vacancies during phase transformation. Dislocations disrupt local charge symmetry, generating equivalent dipoles that synergize with sulfur vacancies to enhance dipole polarization. To semi-quantitatively validate this electronic structure transition, first-principles calculations were performed. The computed band structures (Fig. 1e, f) reveal that 1T-MoS_2_ exhibits gapless metallic character, consistent with its high intrinsic conductivity, whereas 2H-MoS_2_ possesses a band gap of approximately 1.8 eV, confirming its semiconducting nature. These results theoretically substantiate the semiconductor-to-metal transition achieved via electric field postprocessing. Density of states (DOS) analysis (Fig. 1g) indicates that electric field induced *d*-orbital electron migration in Mo ions shifts the *d*-band center, inducing lattice distortion. This confirms that the conductivity and surface chemical properties are predominantly governed by Mo *d*-orbital electrons, validating the feasibility of property modulation through controlled *d*-orbital occupancy. Furthermore, work-function calculations (Fig. 1h) show that the work function of the 1T phase (4.980 eV) exceeds that of the 2H phase (4.844 eV), indicating enhanced charge confinement at the 2H/1T interface and a higher electron escape barrier [28]. Finally, we mixed different sample powders with an elastomer to fabricate electromagnetic wave absorption patches for communication devices. These patches were then applied to high‑radiation regions on mobile phone motherboards. The patch made from the untreated sample reduced the electric field radiation from approximately 900 to about 100 V m^−1^, whereas the patch prepared from the sample treated at 6 V for 10 min reduced the radiation from approximately 900 to around 40 V m^−1^ (Figs. 1i and S6). Collectively, through multi-dimensional analysis, from macroscopic experiments to atomic orbitals, and from theoretical calculations to experimental validation, this study establishes a comprehensive mechanistic framework: the DC electric field drives charge transfer and modulates ion coordination, inducing changes in the Mo coordination environment and orbital hybridization, while concurrently introducing tunable defects such as vacancies and dislocations during the phase transition. This framework provides a robust physicochemical foundation for the performance optimization phenomena observed throughout this work.

### Electrical Field Postprocessing Regulates MoS_2_ Phase Transformation, Interface and Defects

**Fig. 2:**
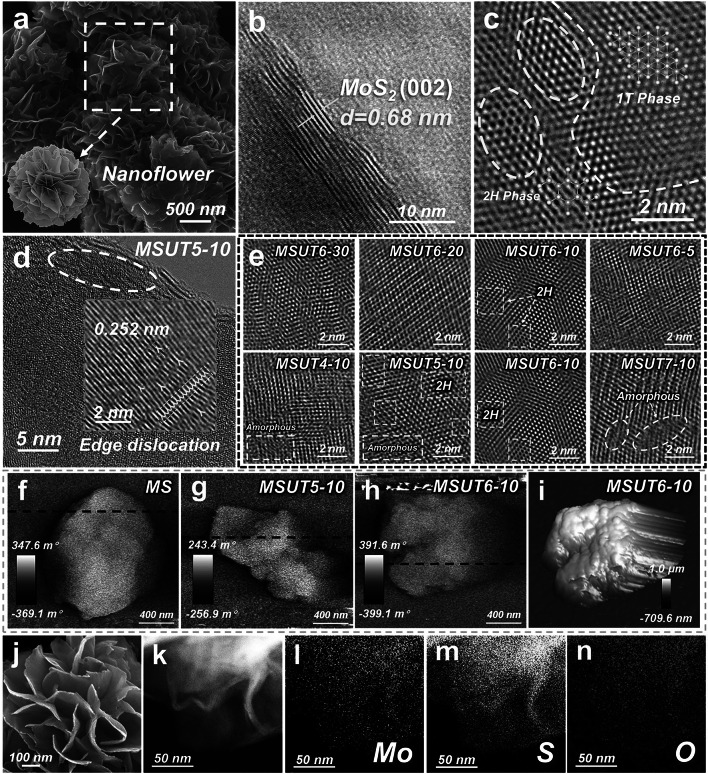
**a**, **j** SEM and TEM images of MS; **b**, **c** HRTEM images of MSUT6-10; **d** HRTEM image of MSUT5-10; **e** HRTEM images of all samples; **f**–**h** EFM images of the samples; **i** AFM image of MSUT6-10; **k–n** elemental mapping images of Mo, S, and O

The microstructure, crystal structure, and surface potential evolution of MoS_2_ before and after electric field treatment were systematically characterized using multiple techniques. Scanning electron microscopy (SEM) reveals that electric field postprocessing did not alter the overall morphology; all samples possess flower-like microspheres (Figs. 2a, j and S7–S9) self-assembled from curved nanosheets, with diameters of approximately 1–2 µm. Transmission electron microscope (TEM) imaging of the edge regions revealed semi-transparent features (Fig. S10), confirming that the nanosheets are only a few atomic layers thick. High-resolution TEM (HRTEM) images displayed clear lattice fringes (Fig. 2b) with an interlayer spacing of approximately 0.68 nm, confirming the layered stacking nature. As shown in Fig. 2c, both trigonal prismatic and octahedral phases coexist within the same field of view, with distinct grain boundaries serving as active sites for interfacial polarization [29]. Figure 2d clearly presents dislocations induced by the electric field during the phase transition in sample MSUT5-10, serving as structural evidence for dislocation-induced polarization. Figure 2e systematically illustrates the lattice evolution under different voltages and processing durations. With increasing voltage and extended time, HRTEM revealed a non‑monotonic trend in lattice integrity and local ordering, first enhancing, reaching an optimum, and then slightly declining, visually reflecting the progression of the 2H to 1T-phase transition [30]. Under the optimal condition of 6 V for 10 min, both lattice ordering and 1T-phase content peaked simultaneously, indicating an optimal balance between phase ratio and structural integrity. These results demonstrate that precise control over electric field parameters enables effective regulation of the phase transition and microstructural evolution in MoS_2_, establishing a structural foundation for the subsequent optimization of its macroscopic electromagnetic wave absorption performance.

To elucidate the charge behavior under electric field modulation and its contribution to polarization effects, we employed electrostatic force microscopy (EFM) to map the surface potential distribution of samples featuring abundant 2H/1T interface (Figs. 2f–h and S11). The EFM phase images of all samples exhibit pronounced bright–dark contrast, confirming that electric field postprocessing induces inhomogeneous charge accumulation and the formation of localized space-charge regions on the sample surface. This non-uniform charge distribution serves as a key driving force for interfacial polarization, correlating with the relaxation features observed in the dielectric spectra (Fig. S12). The measured phase spans are 716.7, 500.3, and 790.7 m, respectively. Among these, sample MSUT5-10 displays the smallest phase span, indicating weaker interactions among surface dipole states and a higher propensity for polarization reorientation under an external electric field, thereby favoring polarization loss. Furthermore, atomic force microscopy (AFM) imaging (Fig. 2i) reveals a certain surface roughness, which further increases the effective specific surface area and density of active sites. HRTEM-EDS elemental mapping (Fig. 2k–n) confirms the homogeneous distribution of Mo, S, and O signals throughout the nanoflower microspheres (Fig. 2l–n), demonstrating excellent chemical uniformity. Collectively, these results confirm that three-dimensional nanoflower-like MoS_2_ was successfully synthesized. Microscopic characterization reveals that electric field postprocessing induces abundant 2H/1T interface, along with surface charge rearrangement and localization; concurrently, the increased 1T-phase content optimizes the internal conductive network, synergistically enhancing both conductive loss and interfacial polarization response. These mesoscopic and microscopic regulatory mechanisms collectively optimize the complex permittivity of the material, providing the physical foundation for achieving broadband and strong absorption performance.

**Fig. 3:**
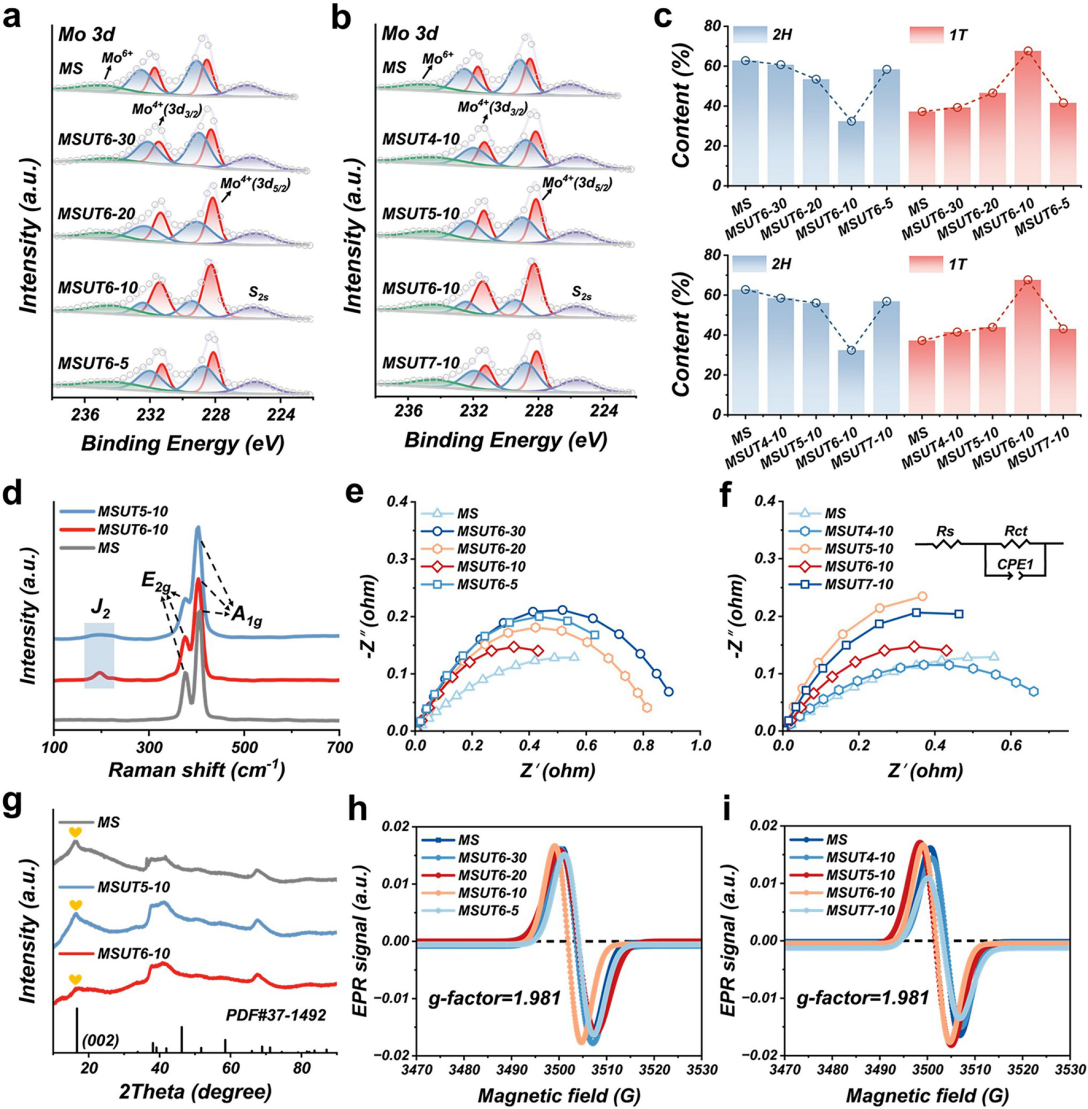
**a**, **b** XPS spectra of Mo 3*d* for all samples; **c** phase contents of 2H-MoS_2_ and 1T-MoS_2_ in all samples; **d** Raman spectroscopy of the samples; **e**, **f** Nyquist plots of the samples; **g** XRD patterns of the samples; **h**, **i** EPR spectra of the samples

To gain deeper insights into the modulation mechanism of electric field postprocessing on the electronic states and crystal structure of MoS_2_, this study comprehensively employed multiple spectroscopic and electrochemical characterization techniques to systematically analyze the evolution of electronic structure in samples subjected to different voltages and processing durations. The work reveals the underlying physicochemical mechanism of electric field induced phase transition from multiple perspectives, including electron configuration, lattice vibration, and defect states. X-ray photoelectron spectroscopy (XPS) analysis (Figs. 3a, b and S13–S18) shows that the electronic structure of the Mo 3*d* orbitals undergoes significant changes with increasing voltage and processing time. The pristine sample exhibits characteristic peaks at binding energies of 228.5 and 231.7 eV, assigned to the 3*d*_5/2_ and 3*d*_3/2_ orbitals of Mo^4+^ in 1T-MoS_2_. After electric field treatment at 6 V for 10 min, the 1T-MoS_2_ characteristic peaks appear at 228.2 and 231.4 eV, showing a redshift of approximately 0.3 eV. This shift reflects a rearrangement of the local electronic environment around Mo atoms, whose physical origin lies in the electric field induced transformation of Mo coordination from trigonal prismatic (2H phase) to octahedral (1T phase). In the octahedral crystal field, the Mo *d*-orbitals split into lower-energy t_2g_ (*d*_xy_, *d*_xz_, *d*_yz_) orbitals and higher-energy e_g_ (*d*_z_^2^, *d*_x_^2^_-y_^2^) orbitals. In the 1T phase, electrons preferentially occupy the t_2g_ band [31], leading to a notable increase in the density of states near the Fermi level and consequently a reduction in the measured binding energy of core electrons. Notably, the area of the 1T-phase characteristic peaks increases nonlinearly with rising voltage and prolonged processing time, indicating that the electric field driven phase transition is governed by the interplay of interfacial energy and strain energy. Figure 3c quantitatively analyzes the evolution of 1T-phase content under different voltages and processing times. Peak-fitting analysis of XPS spectra reveals a non-monotonic trend: the 1T-phase content first rises and then declines with increasing electric field parameters, reaching a maximum of 67.63% under the condition of 6 V for 10 min. This behavior can be elucidated by an electric field driven phase-transition barrier model. In the initial stage, energy injection drives the transformation from 2H to 1T phase, increasing the 1T-phase proportion. When parameters exceed critical thresholds, sustained energy input induces partial reverse transformation of metastable 1T regions or excessive defect aggregation, resulting in a slight decrease in the apparent 1T-phase content. This trend reveals the dynamic equilibrium between electric field parameters and phase structure, namely, that the regulatory outcome is determined by the phase-transition driving force and the overall energetic stability of the system.

Raman spectroscopy analysis (Figs. 3d and S19) provides structural evidence of the phase transition from the perspective of lattice vibrations. The pristine sample exhibits sharp characteristic peaks at 383 and 408 cm^−1^, corresponding to the E^1^_2_g and A_1_g vibrational modes typical of 2H-MoS_2_. After electric field treatment, the intensity of these peaks decreases markedly and their full width at half maximum increases, indicating disruption of long-range order and reduced lattice symmetry. Concurrently, broad vibrational peaks attributed to the 1T phase appear in the range of 150–250 cm^−1^. Under the optimal condition of 6 V for 10 min, the intensity variation of the J₂ peak clearly reflects the distribution of different stacking-sequence variants of the 1T phase, consistent with the semi-quantitative phase ratio obtained from XPS analysis. Furthermore, electrochemical impedance spectroscopy (EIS) analysis (Fig. 3e, f) reveals the influence of the phase transition on conductive properties from the viewpoint of charge transport kinetics. The decreased diameter of the semicircle in the Nyquist plot indicates a significant reduction in charge-transfer resistance. This phenomenon originates from the substantial formation of the metallic 1T phase, which constructs an efficient electron conduction network within the material, substantially increasing carrier mobility and thus markedly enhancing the intrinsic electrical conductivity, ultimately leading to a significant improvement in conductive loss.

X-ray diffraction (XRD) patterns (Figs. 3g and S20) profoundly reflect the macroscopic evolution of the crystal structure. The reduced intensity and broadening of the (002) diffraction peak primarily stem from the disruption of long-range order and lattice distortion caused by the coexistence of the 2H and 1T phases and the formation of 2H/1T interface during the electric field induced phase transition. Electron paramagnetic resonance (EPR) analysis (Figs. 3h, i and S21) reveals that all samples exhibit an EPR signal at g ≈ 1.981, attributed to sulfur vacancies, with the signal intensity showing a non-monotonic variation with applied voltage or processing time, yet the overall differences remain relatively small. This result indicates that, within the range of electric field parameters investigated in this study, the concentration of sulfur vacancies undergoes dynamic evolution during the phase transition process [32, 33], but the extent of its variation is limited and does not constitute the dominant factor responsible for the differences in electromagnetic wave absorption performance among the samples. In summary, through multi-dimensional correlation analysis of characterization data, this study systematically elucidates that the electric field postprocessing strategy induces lattice distortion via *d*-orbital electron migration while simultaneously constructing 2H/1T interface and defects such as sulfur vacancies. Among these, the 2H/1T interface contribute to interfacial polarization response, sulfur vacancies act as dipole centers enhancing dipole polarization behavior, and the conductive network predominantly formed by the 1T phase enhances conductive loss by improving carrier transport efficiency. The close agreement between the aforementioned multi‑mechanism effects and the observed dielectric relaxation behavior confirms the effectiveness of the electric‑field post‑treatment strategy in precisely modulating electromagnetic wave loss and optimizing absorption performance.

### Electrical Field Postprocessing Regulates the Electromagnetic Wave Absorption Performance of MoS_2_

**Fig. 4:**
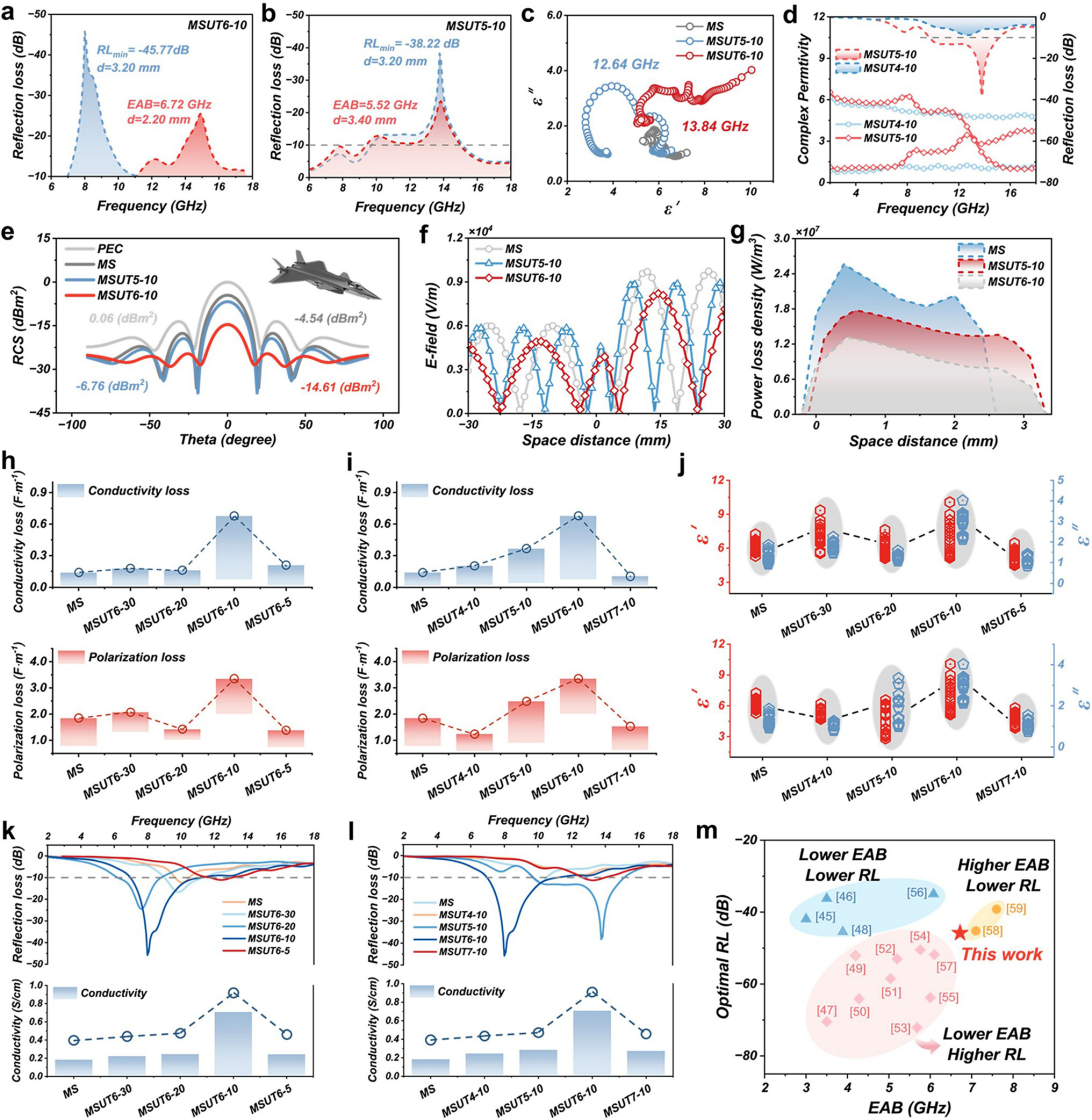
**a**, **b** EWA performance maps of MSUT6-10 and MSUT5-10; **c** cole–cole plots of the samples; **d** comparison of electromagnetic wave absorption performance and electromagnetic parameters between MSUT4-10 and MSUT5-10; **e** RCS plots of the samples; **f**, **g** simulated electric field and power loss density distributions of the samples; **h**, **i** contributions of conductive loss and polarization loss; **j** complex permittivity of the samples; **k**, **l** RL_min_ and electrical conductivity of the samples; **m** EWA properties of reported MoS_2_-based composite materials

To systematically elucidate the optimization mechanism of electric field postprocessing on the EWA performance of MoS_2_, we combined electromagnetic measurements with simulations to analyze the precise modulation of wave absorption characteristics by electric field parameters. The performance maps in Figs. 4a, b and S22–S25, show that under the treatment condition of 5 V for 10 min, the sample exhibits a minimum reflection loss (RL_min_) of − 38.22 dB at a thickness of 3.2 mm and an EAB of 5.52 GHz at 3.4 mm. Under the optimal condition of 6 V for 10 min, the EAB expands to 6.72 GHz, accompanied by a significantly reduced RL_min_ of − 45.77 dB, indicating that the electric field postprocessing strategy enables precise optimization of the material’s wave absorption performance. Cole–Cole analysis based on the Debye relaxation model (Figs. 4c and S26) further reveals the evolution of polarization mechanisms. The Cole–Cole plot of the pristine sample exhibits small and irregular semicircular arcs, indicating limited polarization mechanisms and dispersed relaxation processes. In contrast, electric field treated samples display notably improved dielectric relaxation behavior. The Cole–Cole plot of sample MSUT6-10 shows numerous semicircular arcs with relatively large radii, exhibiting distinct multiple-relaxation characteristics.

The dielectric loss tangent spectra derived from the evolution of electromagnetic parameters (Fig. S27) further quantify the frequency response of different loss mechanisms. The introduction and synergistic operation of these strong polarization loss mechanisms collectively constitute a multi-polarization relaxation system across a broad frequency range [34, 35], underpinning the realization of broadband and high-efficiency electromagnetic wave energy dissipation. Notably, the electromagnetic parameter spectra of samples MSUT4-10 and MSUT5-10 (Fig. 4d) reveal that despite their similar electromagnetic parameters, MSUT5-10 exhibits distinct dielectric relaxation peaks at 8–15 GHz. This phenomenon can be attributed to the modulation effect of dislocations on localized electrons: the strain field around the dislocation core disrupts the local symmetry of charges, inducing the formation of equivalent dipoles, which in turn further enhances the dipole polarization response of the system. This inference directly corroborates the dislocation arrays observed in the HRTEM images of sample MSUT5-10, providing microstructural evidence for the dislocation-enhanced polarization mechanism.

Full-wave simulations using CST Microwave Studio (Figs. 4f, g and S28–S31), based on the finite-difference frequency-domain method, intuitively reveal the dissipation behavior of electromagnetic energy within the material. The simulation results show that at 8.08 GHz with a matching thickness of 3.20 mm, sample MSUT6-10 achieves optimal impedance matching, with more concentrated and uniform distributions of electric and magnetic field energy inside the material, indicating that electromagnetic energy can be coupled into the material more efficiently and dissipated. Although its local power loss density peak is not the highest, the overall EWA performance is optimized under this parameter combination, as evidenced by its excellent broadband absorption capability [36–38]. Fitting and decomposition of electromagnetic parameters based on Debye theory (Figs. 4 h, i and S32) indicate that the contribution of conductive loss first increases and then decreases with increasing 1T-phase content. This phenomenon reflects the existence of an optimal processing window for constructing the conductive network: the conductive loss contribution reaches a maximum after the formation of a percolation threshold network; however, excessively high voltage or prolonged processing time may cause a slight decrease in conductive loss efficiency due to lattice distortion or enhanced defect scattering. Moreover, with increasing frequency, the dominant loss mechanism in samples subjected to different voltages and processing times shifts from conductive loss to polarization loss [39, 40]. Furthermore, the 2H/1T interface induced by electric field postprocessing contribute to interfacial polarization through space-charge accumulation [41, 42], while sulfur vacancies and dislocations respectively generate localized strong electric fields via charge trapping and induce the formation of equivalent dipoles, collectively enhancing the dipole polarization loss of the system. The spectral analysis of intrinsic electromagnetic parameters (Fig. 4j) explains the origin of the performance improvement from the perspective of dielectric physics. After electric field postprocessing, pronounced relaxation peaks appear in the imaginary part of the complex permittivity within the 8–14 GHz range. These peaks corroborate the Cole–Cole analysis and collectively confirm the important role of polarization relaxation mechanisms in the electric field postprocessing modulation of electromagnetic wave absorption performance. The λ/4 model further accounts for the shift of the RL peak toward lower frequencies with increasing coating thickness (Fig. S33) [43, 44]. Transmission line theory analysis reveals that MSUT6-10 exhibits excellent impedance matching characteristics in the 8–18 GHz range, with its normalized input impedance maintained within the ideal interval of 0.8–1.2, ensuring efficient coupling of electromagnetic waves into the material interior.

RL and conductivity analyses (Fig. 4k, l) demonstrate that following electric field postprocessing, the electromagnetic wave absorption performance is significantly enhanced. Sample MSUT6-10 exhibits the highest intrinsic electrical conductivity, which not only directly strengthens the conductive loss capability but also synergizes with polarization characteristics to optimize broadband impedance matching, thereby providing critical conditions for precisely tailoring electromagnetic wave absorption performance. Furthermore, radar cross section (RCS) analysis (Figs. 4e and S34–S35) reveals that, compared with a perfect electric conductor, MSUT6-10 exhibits substantially reduced scattering signals, with an RCS value of − 14.61 dB m_2_ at 0° incidence that remains stable over a wide angular range of ± 90°, thereby validating its excellent wave absorption capability from the perspective of stealth effectiveness. Additionally, Fig. 4m and Tables S1, S2 summarize the EAB and RL_min_ of the MoS_2_-based and other types of absorbing materials reported in this work and in recent literature [45–59]. Collectively, the electric field postprocessing strategy optimizes the wave absorption performance of MoS_2_ through multi-path synergy: the 1T phase dominates conductive loss by optimizing the conductive network, 2H/1T interface contribute to interfacial polarization response, and sulfur vacancies together with dislocation-induced equivalent dipoles synergistically enhance dipole polarization. The synergistic interplay of these microscopic mechanisms enables the material to achieve broadband and strong absorption at the macroscopic level, providing a theoretical foundation for the electric field design of EWA materials.

### Electrical Field Postprocessing Regulates the Electromagnetic Wave Absorption Mechanism of MoS_2_

**Fig. 5:**
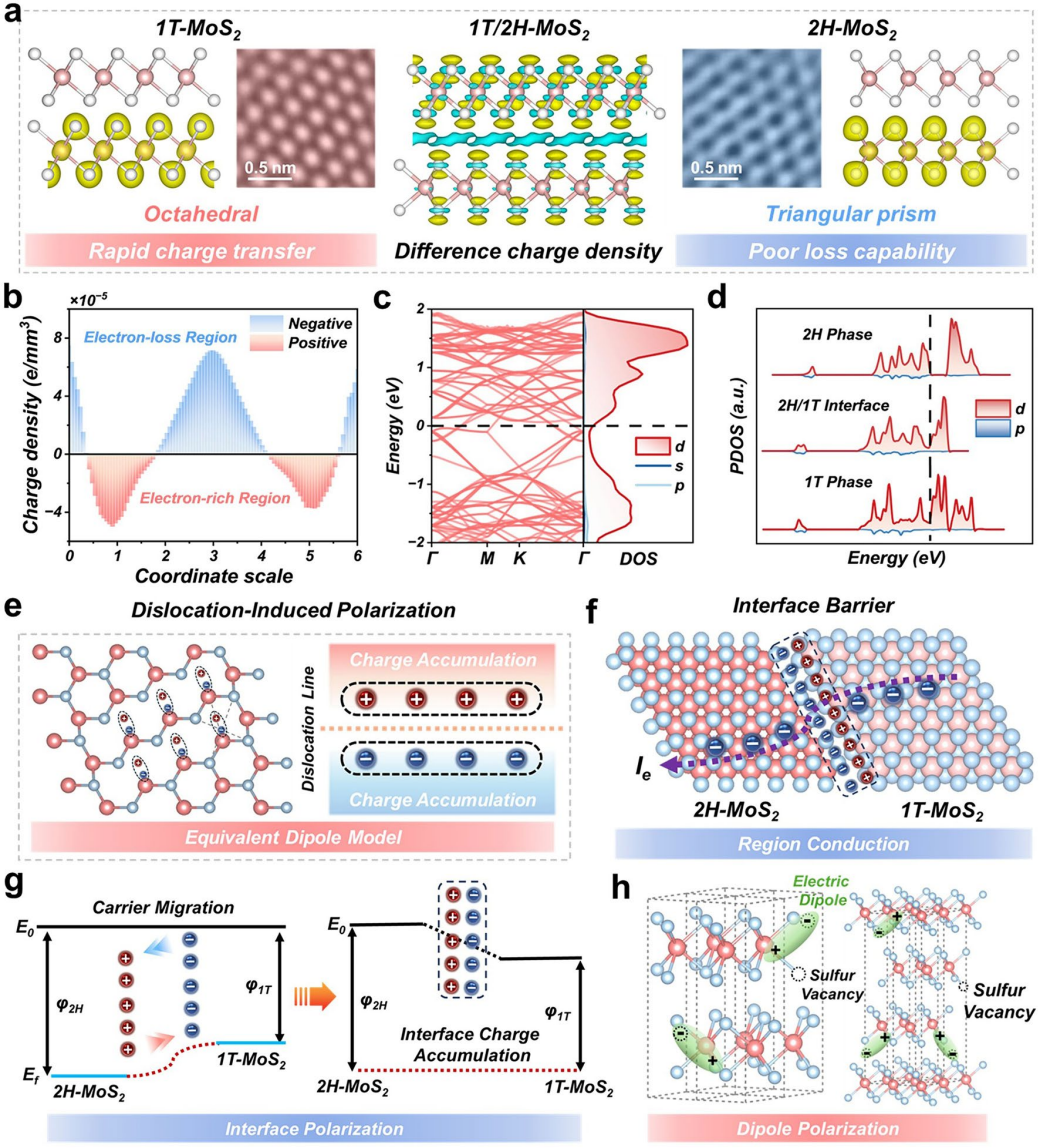
**a** Difference charge density at the 2H/1T interface; **b** one-dimensional profile of the charge density difference; **c** band structure of the 2H/1T interface; **d** DOS of the 2H phase, 1T phase, and 2H/1T interface; **e** schematic illustration of dislocation-induced polarization; **f** conductive loss mechanism; **g** interfacial polarization loss mechanism; **h** dipole polarization loss mechanism

To further elucidate the microscopic physical origin of the observed performance differences, first-principles calculations were employed, providing theoretical support for the proposed synergistic mechanism [60]. Difference charge density analysis (Fig. 5a) reveals the formation of electron depletion regions between the layers of the 2H and 1T phases, along with electron accumulation regions near the sulfur atoms on both sides of the interface. This “central depletion–lateral accumulation” charge distribution pattern confirms significant charge separation and rearrangement at the 2H/1T interface, driving charge accumulation that triggers interfacial polarization. Furthermore, the alternating distribution of electron depletion and electron accumulation regions in the one-dimensional charge density difference profile (Fig. 5b) further reveals the relative intensity of directional charge transfer between the 2H and 1T phases. Subsequently, Fig. 5c, d presents the band structure of the 2H/1T interface model and the partial density of states (PDOS) of the 1T phase, 2H phase, and the interface region. The band structure of the 2H/1T interface exhibits gapless characteristics, with multiple bands crossing the Fermi level, indicating metallic behavior at the interface. This result originates primarily from the hybridization and reconstruction of Mo *d*-orbitals and S *p*-orbitals at the interface, confirming from an electronic energy level perspective the substantial modulation effect of the 2H/1T interface on the intrinsic properties of the material.

Therefore, based on the aforementioned experimental characterization and theoretical calculations, this study systematically elucidates the synergistic electromagnetic wave absorption mechanism involving multiple polarization effects induced by electric field postprocessing (Fig. 5e–h). At the atomic scale, DC electric field postprocessing enhances the dipole polarization loss of the material through two synergistic pathways (Fig. 5e, h) [61, 62]. On one hand, the sulfur vacancies generated by electric field induction disrupt the local charge balance of the lattice, forming permanent electric dipole moments that directly contribute to dipole polarization loss. On the other hand, HRTEM results (Figs. S36 and S37) confirm the generation of dislocations in the samples. As regions of lattice distortion, the dislocation cores create tensile strain fields in their surroundings that disrupt the local symmetry of charge distribution, leading to a slight separation between positive and negative charge centers and forming equivalent dipole arrays uniformly arranged along the dislocation lines. This ordered arrangement increases the net charge on opposite sides of the dislocation lines, making the equivalent dipoles more prone to relaxation under an applied electric field, thereby further enhancing dipole polarization loss. Combined with EPR (Fig. 3h, i) and electromagnetic parameter analysis (Fig. 4d), compared to discretely distributed sulfur vacancy dipoles, the dislocation-induced equivalent dipole arrays play a more dominant role in the dipole polarization response owing to their long-range ordering. The two dipole polarization mechanisms exhibit complementary synergy in spatial distribution and response characteristics, collectively enhancing the overall dipole polarization capability of the material. This microscopic mechanism is highly consistent with the clearly visible dislocation arrays observed in HRTEM images, providing experimental evidence for the dislocation-dominated enhancement of dipole polarization. We performed quantitative analysis of the local strain field around dislocations using geometric phase analysis (GPA, Fig. S38). Distinct tensile/compressive strain reversal regions are observed, corresponding to localized dislocations in the lattice. These reversal regions induce a built‑in electric field at their interfaces, leading to pronounced separation of positive and negative charges and consequently enhanced polarization behavior [63, 64]. Furthermore, we calculated the charge density difference in the dislocation region using DFT, which also reveals a clear separation of positive and negative charges, consistent with the above findings.

Furthermore, during the electric field induced phase transition, the dislocation defects introduced by lattice distortion serve a dual role. On one hand, dislocations facilitate charge trapping during transport within the lattice, inhibiting radial charge transport at grain boundaries and thereby altering the charge transport pathway. This reduces the charge transport efficiency of the three-dimensional conductive network constructed by the 1T phase, leading to decreased conductive loss. On the other hand, this mechanism simultaneously suppresses the skin effect induced by excessively high 1T-phase content, effectively alleviating the resulting impedance mismatch and thereby enhancing the electromagnetic wave absorption performance (Fig. 5f) [65–67]. The 2H/1T interface, owing to the Fermi level difference between the two phases, induces directional migration of charge carriers and the formation of electron rich zone on both sides of the interface [68, 69], thereby generating interfacial polarization loss (Fig. 5g). In contrast to sample MSUT5-10, which exhibits a relatively low imaginary permittivity and limited overall loss capability, sample MSUT6-10 achieves optimal synergy among 2H/1T interface, defects, and the three-dimensional conductive network through precise electric field modulation. While retaining interfacial polarization and dipole polarization, it effectively suppresses the impedance mismatch arising from excessive 1T-phase content, thereby achieving synergistic optimization between polarization and conduction.

## Conclusion

We introduce a DC electric field postprocessing strategy to regulate the microstructure and electromagnetic wave absorption of MoS_2_. By controlling *d*-orbital electron migration, this method triggers the 2H to 1T-phase transition and generates dislocations. The increased 1T-phase content strengthens conductive loss through an optimized three-dimensional conductive network, while the Fermi level mismatch at 2H/1T interface creates electron accumulation regions that induce polarization loss. Additionally, charge separation along dislocation lines forms ordered equivalent dipole arrays, markedly boosting polarization response. MSUT6-10 achieves an effective absorption bandwidth of 6.72 GHz at 2.20 mm, a 440% improvement over the pristine sample. This work reveals the dipole polarization behavior arising from dislocation-induced equivalent dipoles and provides a feasible strategy for the design of “smart absorbing materials” with externally tunable fields.

## Supplementary Information

Below is the link to the electronic supplementary material.

Supplementary file1 (DOCX 17408 KB)
