# Supplementary material for: Polymorphic Transformation and Dislocation Regulation in MoS2 Enabled by Electric Field Postprocessing for Enhanced Electromagnetic Wave Absorption
Source: Nanomicro Lett. 2026 Aug 12;19:32. doi: 10.1007/s40820-026-02334-1 (PMC13469053; doi:10.1007/s40820-026-02334-1)
Supplement: Supplementary file 1 [file 40820_2026_2334_MOESM1_ESM.docx]

Supplementary Information for

**Polymorphic Transformation and Dislocation Regulation in MoS_2_ Enabled by Electric Field Postprocessing for Enhanced Electromagnetic Wave Absorption**

Xiangdong Wang^1,2, #^, Zijing Li^1, #^, Shengchong Hui^1^, Hongjing Wu^1,^ *, Jiaming Wen^1^ and Limin Zhang^1, 2,^ *

^1^ State Key Laboratory of Porous Metal Materials, School of Physical Science and Technology, Northwestern Polytechnical University, Xi’an 710072, P. R. China

^2^ Shenzhen Research Institute of Northwestern Polytechnical University, Sanhang Science &Technology Buliding, No. 45th, Gaoxin South 9th Road, Nanshan District, Shenzhen 518057, P. R. China

^#^Xiangdong Wang and Zijing Li contributed equally to this work.

* Corresponding authors. E-mail: [wuhongjing@nwpu.edu.cn](mailto:wuhongjing@nwpu.edu.cn) (Hongjing Wu); [liminzhang@nwpu.edu.cn](mailto:liminzhang@nwpu.edu.cn) (Limin Zhang)

**S1 Supplementary Text**

**S1.1 Preparation of MSUTX-X**

This DC voltage stabilizing electric field device mainly consists of the following components: a 10*10*3 mm graphite electrode as the block-shaped cathode, a 5*10*0.2 mm metal platinum electrode as the sheet-shaped anode, and a 100 ml electrolytic cell as the device container. The distance between the electrodes is 18 mm. The input voltage of the power supply (DC current, 0~36 V, 0~6 A).

**S2 Characterization Methods**

**S2.1 Materials Characterization**

The crystal structure of the samples was characterized by a Bruker D8 Advance X-ray diffractometer (XRD, Co Kα radiation, 3 kW) with a data collection range of 10° to 90° (2θ). Raman spectra of the samples were obtained using a Renishaw in-vivo confocal Raman microscope (excitation wavelength 514.5 nm) to analyze the molecular vibration modes and phase structure. The molecular structure and functional group types of the samples were characterized by a Fourier transform infrared spectrometer (Nicolet iS 10). The micro-surface morphology of the samples was observed by a scanning electron microscope (SEM, Zeiss Sigma 300, Germany). The precise lattice configuration and elemental distribution of the samples were analyzed by a transmission electron microscope (TEM, FEI Talos F200X) equipped with an X-ray energy dispersive spectrometer (EDS, Oxford). Before testing, the samples were dispersed in ethanol, ultrasonically treated to ensure uniform dispersion, and then the dispersion was dropped onto a copper grid and dried for SEM and TEM observation. The surface roughness of the samples was characterized by an atomic force microscope (AFM, Bruker Dimension ICON), and the surface charge distribution was analyzed using its electrostatic force microscopy (EFM) mode. The elemental chemical states and electronic structure of the samples were analyzed by an X-ray photoelectron spectrometer (XPS, Kratos Axis Ultra DLD). The defect concentration in the samples was characterized by an electron paramagnetic resonance spectrometer (EPR, Bruker A 300, 300 K). Electrochemical impedance spectra (EIS) were collected in the frequency range of 0.01 Hz to 100 kHz by applying a 10 mV AC voltage amplitude at the open circuit potential using an electrochemical workstation (Shanghai Chenhua, chi760e).

**S2.2 Electromagnetic Measurement**

After mixing the sample with paraffin, it was pressed into a custom coaxial ring (𝑑_𝑜𝑢𝑡_= 7.00 mm, 𝑑_𝑖𝑛_ = 3.04 mm). The electromagnetic parameters of the sample were measured within the microwave frequency range of 2-18 GHz using a network vector analyzer (NVA, Agilent MS46322B, Japan). Usually, the reflection loss (RL) is used to evaluate the electromagnetic wave absorption performance of the absorber. If the reflection loss value is less than -10 decibels (indicating that 90% of the energy is absorbed), then the electromagnetic wave absorption at that frequency point is considered to be effective. According to the transmission line theory, the reflection loss can be calculated using the following formula [S1]:

$RL=20 log\left| \left( Z_{in}-Z_{O} \right)/\left( Z_{in}+Z_{O} \right) \right|$	(S1)

$Z_{in}=Z_{0}\sqrt{\frac{\mu_{r}}{\varepsilon_{r}}} tanh\left( j\frac{2\pi fd}{c}\sqrt{\mu_{r}\varepsilon_{r}} \right)$	(S2)

Among them, Z_0_, Z_in_, d and c refer to the impedance of free space, impedance of the absorber, the thickness of absorber, and velocity of light, respectively.

**S2.3 Radar Cross Section (RCS) Simulation**

The RCS simulations were performed in CST software based on the far-field response. Specifically, the model consisted of an upper wave absorption layer and a lower perfect electrical conductor (PEC) layer, with thicknesses of 3.2 mm and 1.0 mm respectively. The length and width of each layer were designed to be 100 mm. The model was placed on the XOY plane, and a plane wave was used as the incident wave propagating along the negative z-axis. The polarization direction was along the z-axis. Additionally, the boundary conditions were set as open boundaries, and the selected field monitoring frequency was 8.08 GHz, which was derived from the frequency corresponding to the optimal RL_min_ of MSUT6-10.

The RCS performance of the sample is evaluated using the following formula [S2]:

$\mathrm{RCS}\left( \mathrm{dB}m^{2} \right)=10log\left( \frac{4\pi S}{\lambda^{2}}\left| \frac{E_{S}}{E_{i}} \right|^{2} \right)$	(S3)

Where S is the area of the layer of the simulated plate (100 mm^2^🞨100 mm^2^), and λ is the EM wave wavelength. Es and Ei represent the electric field intensity of scattered EM waves and incident EM waves, respectively.

**S2.4 Conduction loss (ε_c_″) and Polarization Loss (ε_p_″) Calculation**

The 𝜀_c_″ and 𝜀_p_″ can be calculated into the following Eqs. [S3]:

$\varepsilon''=\varepsilon_{c}''+\varepsilon_{p}''=\frac{\varepsilon_{s}-\varepsilon_{\infty}}{1+\omega^{2}\tau^{2}}\omega\tau+\frac{\sigma}{\omega\varepsilon_{0}}$	(S4)

$\varepsilon_{p}''=\frac{\varepsilon_{s}-\varepsilon_{\infty}}{1+\omega^{2}\tau^{2}}\omega\tau$	(S5)

The *ε*_s_, *ε*_∞_, *τ*, and *σ* represent the static and infinite frequency dielectric constant, Debye relaxation time and electrical conductivity, respectively.

**S2.5 Density Functional Theory (DFT) Calculation**

This paper conducts a systematic study on the electronic structure and surface properties of materials based on the first-principles calculation method of density functional theory (DFT), using the Vienna ab initio simulation package (VASP). The generalized gradient approximation (GGA) framework's Perdew-Burke-Ernzerhof (PBE) exchange-correlation functional is adopted to describe the exchange-correlation interaction between electrons. The calculation content mainly includes the following aspects: differential charge density analysis to reveal the charge transfer behavior during the bonding process between atoms; work function calculation to evaluate the electron escape ability of the material surface; band structure and projected density of states analysis to clarify the electronic energy level distribution and orbital hybridization characteristics of the system. The structural modeling is based on the crystal information file (cif) of the material, and the high-symmetry points and their closed paths within the Brillouin zone are determined through the Seekpaths path analysis tool to ensure the completeness and accuracy of the band structure calculation. Before the self-consistent calculation and density of states analysis, the unit cell structure is fully optimized geometrically. During the optimization process, the conjugate gradient method is used for iterative solution, and the energy convergence threshold is set to 1×10^-8^ eV to ensure that the system reaches the ground state stable configuration. The Brillouin zone integration adopts the Monkhorst-Pack method, and the K-point grid density is set to 6×6×1 to balance the calculation accuracy and efficiency. To avoid the interlayer interaction introduced by the periodic boundary condition, a 15 Å vacuum layer is set in the vertical direction (z-axis) to effectively isolate adjacent periodic images.

**S2.6 Electromagnetic Radiation Detection Experiment**

Mix MSUT6-10 and polyvinylidene fluoride in a 1:6 mass ratio in 25 ml of *N*, *N*-dimethylformamide to prepare an EWA patch with a thickness of approximately 2 mm. Then, cover the surface of the mobile phone motherboard (the part that receives and transmits signals) with it. Record the dynamic electric field radiation values using an electromagnetic radiation detection instrument (QA062RU). Additionally, tests were conducted on the original sample MS and the mobile phone without the EWA patch.

**S2.7 Working Principle of the Electromagnetic Radiation Detector**

Three independent probes at the tip of the detector convert electromagnetic field energy in the environment into weak alternating electrical signals. These signals are then processed through filtering, amplification, and detection circuits to obtain a stabilized root‑mean‑square value. After calibration, the result is displayed as the electric field strength in volts per meter (V m^-1^).

**S2.8 Electric Field Distribution Inside the Electrolytic Cell Simulated Using Comsol.**

A direct‑current electric field was established using a metal foil (5*10*0.2 mm) as the positive electrode and a graphite electrode (10*10*3 mm) connected to ground. A cylindrical simulation cell was constructed to contain the liquid electrolyte. The two electrodes were placed at the center of the cylinder with a separation distance of 18 mm. Boundary conditions were set such that the outer cylindrical shell was electrically insulating, while the interior of the cylinder was filled with the liquid medium (sodium chloride solution). Charge conservation was enforced throughout the interior except at the boundaries. A mesh with an extremely fine element size was then generated, and the simulation was performed at a frequency of 0 Hz.

**S3** **Supplementary Figures**

**Fig. S1 a**-**d** Electric field distribution under different voltages

**Fig. S2** Simulated current density under different voltages

**Fig. S3 a**, **b** Temperatures of the samples under different voltages and processing times, **c** MoS₂ at 35 °C under the present experimental conditions

**Fig. S4 a**,**b** Electromagnetic parameters and wave absorption performance of MoS_2_ at 35°C in the present study

**Fig. S5 a**-**d** Graphite and platinum electrodes after electric-field treatment

**Fig. S6** Electromagnetic patch test. **a**-**e** Fabrication and presentation of the electromagnetic wave absorption patch. The powder and polyvinylidene fluoride were dissolved and dispersed in DMF to form a paste, which was then poured into a petri dish and dried at 50 °C. Its thickness was measured to be 2 mm, with a width of approximately 70 mm and a length of approximately 100 mm. **f**, **g** Photos of electromagnetic radiation detection with and without the electromagnetic wave absorption patch

**Fig. S7 a**-**d** Scanning electron microscope (SEM) images of the samples and corresponding energy spectrum analysis diagrams, corresponding to Mo, S, and O respectively

**Fig. S8 a**-**d** Scanning electron microscope (SEM) images of the samples under different treatment times, along with corresponding energy spectrum analysis diagrams, corresponding to Mo, S, and O respectively

**Fig. S9 a**-**d** Scanning electron microscope (SEM) images of the samples under different voltages, along with corresponding energy spectrum analysis diagrams, corresponding to Mo, S, and O respectively

**Fig. S10** The TEM image of the edge area of the sample

**Fig. S11 a**, **b** Atomic Force Microscopy (AFM) images of samples MS and MSUT5-10, **c** Charge distribution of samples MS, MSUT5-10, and MSUT6-10

**Fig. S12 a**, **b** Electromagnetic parameter spectra of the samples under different voltages and different treatment times, **c**, **d** Imaginary part of electromagnetic parameters under different voltages and processing times

**Fig. S13 a**-**c** XPS spectra of C1s, O1s, and S2p for the sample MS

**Fig. S14 a**-**d** XPS spectra of C 1*s* for samples under different voltages, **e**-**h** XPS spectra of C 1*s* for samples under different treatment times

**Fig. S15 a**-**d** XPS spectra of O 1*s* for samples under different voltages, **e**-**h** XPS spectra of O 1*s* for samples after different treatment times

**Fig. S16 a**-**d** XPS spectra of S 2*p* for samples under different voltages, **e**-**h** XPS spectra of S 2*p* for samples after different treatment times

**Fig. S17 a**, **c** XPS of sample MS after Raman measurement and of the as‑prepared sample (without Raman measurement), **b**, **d** XPS spectra of sample MSUT6‑10 after Raman measurement and those directly measured (without Raman measurement)

**Fig. S18** 1T phase contents of samples MS and MSUT6‑10 measured after Raman testing and directly (without Raman testing)

**Fig. S19 a**, **b** Raman spectra of the samples under different voltages and different treatment times

**Fig. S20 a**, **b** XRD patterns of the samples under different voltages and different treatment times

**Fig. S21** **a** Unfitted EPR signals of the samples at different times, **b** Unfitted EPR signals of the samples at different voltages

**Fig. S22 a**-**d** Electromagnetic parameters of the sample MS, Cole-Cole diagram, electromagnetic wave absorption performance, dielectric loss tangent spectrum

**Fig. S23 a**-**d** Electromagnetic parameters of the samples under different treatment times, **e**-**h** Electromagnetic wave absorption performance of the samples under different treatment times

**Fig. S24 a**-**d** Electromagnetic parameters of the samples under different voltages, **e**-**h** Electromagnetic wave absorption performance of the samples under different voltages

**Fig. S25 a**, **b** Effective absorption bandwidth of the samples under different voltages and different treatment times

**Fig. S26 a**-**h** Cole-Cole plots of the samples under different voltages and different treatment times

**Fig. S27 a**-**h** Dielectric loss tangent spectra of the samples under different voltages and different treatment times

**Fig. S28** Magnetic field distribution of samples MS, MSUT5-10, and MSUT6-10

**Fig. S29 a**-**h** Three-dimensional electric field distribution maps of the samples under different voltages and different treatment times

**Fig. S30 a**-**h** Three-dimensional magnetic field distribution maps of the samples under different voltages and different treatment times

**Fig. S31 a**-**h** Three-dimensional power loss density of the samples under different voltages and different treatment times

**Fig. S32 a**, **b** The fraction of conductive loss as a function of applied voltage and processing duration

**Fig. S33** **a**, **b** RL curves of MSUT6-10 and MS, **c**, **d** Experimental values of RL peak frequency as a function of thickness, along with the simulated matching thicknesses derived from the quarter‑wavelength model, **e**, **f** Impedance matching ratio |Z_in_/Z_0_| of MSUT6-10 and MS

**Fig. S34 a**-**h** RCS of the samples under different voltages and different treatment times, **i**, **j** RCS of the samples' MS and the metal backplate PEC

**Fig. S35 a**-**g** Three-dimensional radar cross-section diagrams of the samples under different voltages and different treatment times, **h**, **i** Three-dimensional radar cross-section diagrams of the sample MS and the metal backplate PEC

**Fig. S36** **a**-**h** HRTEM images of samples treated under different voltages and for different durations

**Fig. S37 a**, **b** Electromagnetic parameters and wave absorption performance of sample MSUT6-10 after annealing at 400 °C for 2 h

**Fig. S38 a**, **b** TEM-based GPA maps of dislocation-free and dislocated regions, **c** Differential charge density of dislocations

**Table S1** Electromagnetic wave absorption performance of MoS_2_-related absorbing devices

| No. | Samples | *RL*_min_/dB | EAB/GHz | Thickness/mm | Reference |
| --- | --- | --- | --- | --- | --- |
| 1 | MoS_2_-C3 | -41.90 | 3.00 | 1.00 | [S4] |
| 2 | MoS_2_/FeS/ Rhe | -36.20 | 3.50 | 2.00 | [S5] |
| 3 | MoS_2_/SiCnw/Si_3_N_4_ | -70.48 | 3.50 | 2.38 | [S6] |
| 4 | MoS_2_ 1T-50% | -45.50 | 3.89 | 3.20 | [S7] |
| 5 | Ti_3_C_2_Tx/MoS_2_ S2 | -52.10 | 4.20 | 3.10 | [S8] |
| 6 | PCMs | -64.10 | 4.28 | 2.10 | [S9] |
| 7 | NiS/MoS_2_/Ti_3_C_2_T_x_ | -58.49 | 5.04 | 2.10 | [S10] |
| 8 | FeMoS-SWCNTs | -53.01 | 5.20 | 2.00 | [S11] |
| 9 | MBene/MoS_2_ | -72.13 | 5.68 | 1.70 | [S12] |
| 10 | MoS_2_/CoS_2_/VN | -50.48 | 5.76 | 2.02 | [S13] |
| 11 | C@MoS_2_ | -63.80 | 6.00 | 2.10 | [S14] |
| 12 | NDC/ MoS_2_ | -34.94 | 6.08 | 3.00 | [S15] |
| 13 | Fe-Mo-S/CFP | -51.82 | 6.10 | 1.64 | [S16] |
| 14 | MoS_2_/Ti_3_C_2_ MXene | -45.20 | 7.10 | 3.00 | [S17] |
| 15 | MoS_2_ nanoparticles | -39.2 | 7.60 | 3.00 | [S18] |
| 16 | MSUT6-10 | -45.77 | 6.72 | 2.20 | **This work** |

**Table S2** The samples of this study and other types of absorbing materials

| No. | Samples | *RL*_min_/dB | EAB/GHz | Thickness/mm | Reference |
| --- | --- | --- | --- | --- | --- |
| 1 | CoNi@SnO_2_@Sn | -62.29 | 5.40 | 1.26 | [S19] |
| 2 | CoNiMn@C | -30.10 | 5.80 | 2.00 | [S20] |
| 3 | CoNi@NC-CoNi@CNTs-3 | -59.92 | 5.60 | 1.87 | [S21] |
| 4 | Fe-N-RGO | -74.50 | 7.05 | 2.00 | [S22] |
| 5 | (CrMnFeNiCu)_3_O_4_ | -50.70 | 4.70 | 1.94 | [S23] |
| 6 | E/Z-3-700 | -58.81 | 6.39 | 1.97 | [S24] |
| 7 | CNTs/CNF | -68.20 | 5.50 | 2.70 | [S25] |
| 8 | (NiFeCuCoMn)Se_2_ | -38.54 | 3.75 | 1.60 | [S26] |
| 9 | Ni/MXene | -64.70 | 7.20 | 1.50 | [S27] |
| 10 | MSUT6-10 | -45.77 | 6.72 | 2.20 | **This work** |

**Supplementary References**

[S1] Z. Zhao, Y. Qing, L. Kong, H. Xu, X. Fan et al., Advancements in microwave absorption motivated by interdisciplinary research. Adv. Mater. **36**(4), 2304182 (2024). <https://doi.org/10.1002/adma.202304182>

[S2] J. Tao, J. Zhou, Z. Yao, J. Wang, L. Xu et al., Multi-field coupled motion induces electromagnetic wave absorbing property regeneration of elastomer in marine environment. Adv. Funct. Mater. **34**(12), 2310640 (2024). <https://doi.org/10.1002/adfm.202310640>

[S3] W. Deng, T. Li, H. Li, J. Abdul, L. Liu et al., MOF derivatives with gradient structure anchored on carbon foam for high-performance electromagnetic wave absorption. Small **20**(26), 2309806 (2024). <https://doi.org/10.1002/smll.202309806>

[S4] J. Wang, Z. Wu, C. Yang, G. Chen, M. Yuan et al., Controllable regulation of MoS_2_ surface atomic exposure for boosting interfacial polarization and microwave absorption. Adv. Funct. Mater. **34**(51), 2409923 (2024). <https://doi.org/10.1002/adfm.202409923>

[S5] L. Jin, X. Liu, Y. Zheng, Z. Li, Y. Zhang et al., Interface polarization strengthened microwave catalysis of MoS_2_/FeS/Rhein for the therapy of bacteria-infected osteomyelitis. Adv. Funct. Mater. **32**(33), 2204437 (2022). <https://doi.org/10.1002/adfm.202204437>

[S6] J. Bai, S. Huang, X. Yao, X. Liu, Z. Huang, Construction of the SiC nanowires network structure decorated by MoS_2_ nanoflowers in porous Si_3_N_4_ ceramics for electromagnetic wave absorption. Chem. Eng. J. **469**, 143809 (2023). <https://doi.org/10.1016/j.cej.2023.143809>

[S7] M. Ning, P. Jiang, W. Ding, X. Zhu, G. Tan et al., Phase manipulating toward molybdenum disulfide for optimizing electromagnetic wave absorbing in gigahertz. Adv. Funct. Mater. **31**(19), 2011229 (2021). <https://doi.org/10.1002/adfm.202011229>

[S8] M. Li, W. Zhu, X. Li, H. Xu, X. Fan et al., Ti_3_C_2_T*_x_*/MoS_2_ self-rolling rod-based foam boosts interfacial polarization for electromagnetic wave absorption. Adv. Sci. **9**(16), 2201118 (2022). <https://doi.org/10.1002/advs.202201118>

[S9] Y. Feng, G. Hai, G. Sun, K. Chen, X. Wang et al., Dual-functional phase change composites integrating thermal buffering and electromagnetic wave absorption *via* multi-interfacial engineering. Adv. Fiber Mater. **7**(6), 1873–1887 (2025). <https://doi.org/10.1007/s42765-025-00585-y>

[S10] M. Chang, Z. Jia, S. He, J. Zhou, S. Zhang et al., Two-dimensional interface engineering of NiS/MoS_2_/Ti_3_C_2_T*_x_* heterostructures for promoting electromagnetic wave absorption capability. Compos. Part B Eng. **225**, 109306 (2021). <https://doi.org/10.1016/j.compositesb.2021.109306>

[S11] Y. Shen, Z. Ma, F. Yan, C. Zhu, X. Zhang et al., Enhanced interfacial polarization loss of FeS/MoS_2_@N-doped carbon sandwich-walled nanotubes enables high-performance electromagnetic wave absorption. Adv. Funct. Mater. **35**(26), 2423947 (2025). <https://doi.org/10.1002/adfm.202423947>

[S12] Y. Wang, J. Chen, B. Li, M. Que, Y. Ma et al., Controllable mesoporous wrinkling design realizes interfacial charge engineering in hierarchical MBene/MoS_2_ heterostructures for enhanced microwave absorption. Chem. Eng. J. **524**, 169237 (2025). <https://doi.org/10.1016/j.cej.2025.169237>

[S13] J. Zhou, D. Lan, F. Zhang, Y. Cheng, Z. Jia et al., Self-assembled MoS_2_ cladding for corrosion resistant and frequency-modulated electromagnetic wave absorption materials from X-band to ku-band. Small **19**(52), 2304932 (2023). <https://doi.org/10.1002/smll.202304932>

[S14] Y. Liu, F. Wang, Y. Wang, B. Hu, P. Xu et al., A combined engineering of hollow and core-shell structures for C@MoS_2_ microcapsules toward high-efficiency electromagnetic absorption. Compos. Part B Eng. **273**, 111244 (2024). <https://doi.org/10.1016/j.compositesb.2024.111244>

[S15] J. Luo, M. Feng, Z. Dai, C. Jiang, W. Yao et al., MoS_2_ wrapped MOF-derived N-doped carbon nanocomposite with wideband electromagnetic wave absorption. Nano Res. **15**(7), 5781–5789 (2022). <https://doi.org/10.1007/s12274-022-4411-6>

[S16] X. Wang, Q. Zheng, L. Wang, W. Jiang, Phase engineering of MoS_2_ *via* Anderson-type polyoxometalate precursor for efficient electromagnetic wave absorption. Adv. Funct. Mater. **35**(33), 2501720 (2025). <https://doi.org/10.1002/adfm.202501720>

[S17] X.T. Kong, L. Zhang, H. Li, X.B. Zhu, 2D/2D Mo_0.74_W_0.26_S_2_/Ti_3_C_2_ MXene for microwave absorption with broad effective absorption band. Appl. Phys. Lett. **123**(2), 021902 (2023). <https://doi.org/10.1063/5.0156401>

[S18] Z. Feng, P. Yang, G. Wen, H. Li, Y. Liu et al., One-step synthesis of MoS_2_ nanoparticles with different morphologies for electromagnetic wave absorption. Appl. Surf. Sci. **502**, 144129 (2020). <https://doi.org/10.1016/j.apsusc.2019.144129>

[S19] M. Huang, B. Li, Y. Qian, L. Wang, H. Zhang et al., MOFs-derived strategy and ternary alloys regulation in flower-like magnetic-carbon microspheres with broadband electromagnetic wave absorption. Nano-Micro Lett. **16**(1), 245 (2024). <https://doi.org/10.1007/s40820-024-01416-2>

[S20] Y. Chen, L. Gai, B. Hu, Y. Wang, Y. Chen et al., Directional three-dimensional macroporous carbon foams decorated with WC_1−_*_x_* nanoparticles derived from salting-out protein assemblies for highly effective electromagnetic absorption. Nano-Micro Lett. **18**(1), 71 (2025). <https://doi.org/10.1007/s40820-025-01920-z>

[S21] Q. Liang, M. He, B. Zhan, H. Guo, X. Qi et al., Yolk–shell CoNi@N-doped carbon-CoNi@CNTs for enhanced microwave absorption, photothermal, anti-corrosion, and antimicrobial properties. Nano-Micro Lett. **17**(1), 167 (2025). <https://doi.org/10.1007/s40820-024-01626-8>

[S22] K. Zhang, Y. Yan, Z. Wang, G. Ma, D. Jia et al., Integration of electrical properties and polarization loss modulation on atomic Fe–N-RGO for boosting electromagnetic wave absorption. Nano-Micro Lett. **17**(1), 46 (2024). <https://doi.org/10.1007/s40820-024-01518-x>

[S23] G. Zhang, R. Zhang, Z. Zeng, B. Liu, Y. Hou et al., Electron delocalization engineering on 2D high-entropy metal oxides for boosting electromagnetic wave absorption. Adv. Funct. Mater. **36**(30), e27135 (2026). <https://doi.org/10.1002/adfm.202527135>

[S24] J. Tao, P.J. Sugumaran, Y. Zhao, C. Wang, U. Jamwal et al., Cascaded built-In electric fields engineering for electromagnetic wave absorption. Adv. Mater. **38**(25), e23404 (2026). <https://doi.org/10.1002/adma.202523404>

[S25] L. Kong, S. Zhang, Y. Liu, H. Wu, X. Fan et al., Hierarchical architecture bioinspired CNTs/CNF electromagnetic wave absorbing materials. Carbon **207**, 198–206 (2023). <https://doi.org/10.1016/j.carbon.2023.03.024>

[S26] Z. Yan, L. Wang, Y. Du, G. Chen, Y. Wu et al., Local charge regulation in selenides *via* high-entropy engineering to boost electromagnetic wave absorption. Adv. Funct. Mater. **35**(17), 2422787 (2025). <https://doi.org/10.1002/adfm.202422787>

[S27] L. Chang, X. Zhang, T. Liu, B. Li, Y. Ji et al., Multifunctional three-dimensional porous MXene-based film with superior electromagnetic wave absorption and flexible electronics performance. Nano-Micro Lett. **18**(1), 184 (2026). <https://doi.org/10.1007/s40820-025-02034-2>
